# Comparative Analysis of Early and Long-Term Outcomes of Patients with Degenerative Lumbar Spine Disease Using the DIAM Stabilizer and Standard Rehabilitation Program: A Preliminary Prospective Randomized Controlled Trial with 1-Year Follow-Up

**DOI:** 10.3390/healthcare11222956

**Published:** 2023-11-13

**Authors:** Adam Druszcz, Maciej Miś, Małgorzata Paprocka-Borowicz, Joanna Rosińczuk, Bogdan Czapiga

**Affiliations:** 1 Department of Neurosurgery, Provincial Specialist Hospital in Legnica, 59-220 Legnica, Poland; dr.druszcz@gmail.com; 2 Department of Neurosurgery, Specialist Hospital in Walbrzych, 58-309 Walbrzych, Poland; mis.neurochirurg@gmail.com; 3 Department of Physiotherapy, Wroclaw Medical University, 55-355 Wroclaw, Poland; malgorzata.paprocka-borowicz@umw.edu.pl; 4 Department of Nursing and Obstetrics, Wroclaw Medical University, 51-618 Wroclaw, Poland; 5 Department of Neurosurgery, 4th Military Clinical Hospital in Wroclaw, 50-981 Wroclaw, Poland; bogdanczapiga@op.pl

**Keywords:** low back pain, sciatica, degenerative lumbar spine disease, neurosurgery, DIAM, conservative treatment, rehabilitation

## Abstract

Low back pain (LBP) is a leading cause of disability and work absenteeism. The cause of LBP may be degeneration of the intervertebral disc. LBP is characterized by considerable variability and tends to develop into chronic pain. Treatment of LBP includes conservative and rehabilitative treatments, surgery, and so-called minimally invasive treatment. One of the most commonly performed procedures is interspinous stabilization using a dynamic interspinous DIAM (device for intervertebral assisted motion) stabilizer. There is still no clear, strong evidence for the effectiveness and superiority of surgical treatment over conservative treatment. This study aimed to compare the early and long-term outcomes of patients with LBP using the DIAM interspinous stabilizer in relation to patients treated conservatively. A group of 86 patients was prospectively randomized into two comparison groups: A (*n* = 43), treated with the DIAM dynamic stabilizer for degenerative lumbar spine disease (mean age = 43.4 years ± SD = 10.8 years), and B (*n* = 43), treated conservatively. Pain severity was assessed using the visual analog scale (VAS), whereas disability was assessed using the Oswestry disability index (ODI). The difference in preoperative and postoperative ODI scores ≥ 15 points was used as a criterion for treatment effectiveness, and the difference in VAS scores ≥ 1 point was used as a criterion for pain reduction. In patients under general anesthesia, the procedure only included implantation of the DIAM system. Patients in the control group underwent conservative treatment, which included rehabilitation, a bed regimen, analgesic drug treatment and periarticular spinal injections of anti-inflammatory drugs. It was found that all patients (*n* = 43) continued to experience LBP after DIAM implantation (mean VAS score of 4.2). Of the 36 patients who experienced LBP with sciatica before the procedure, 80.5% (*n* = 29) experienced a reduction in pain. As for the level of fitness, the average ODI score was 19.3 ± 10.3 points. As for the difference in ODI scores in the pre-treatment results vs. after treatment, the average score was 9.1 ± 10.6. None of the patients required reoperation at 12 months after surgery. There were no statistically significant differences between the two groups in either early (*p* = 0.45) or long-term outcomes (*p* = 0.37). In conclusion, neurosurgical treatment with the DIAM interspinous stabilizer was as effective as conservative treatment and rehabilitation during the one-year follow-up period.

## 1. Introduction

The problem of degenerative lumbar spine disease (DLSD) and intervertebral disc degeneration disease (IDDD) has been considered from the point of view of several disciplines, from orthopedics and surgery through to histology and molecular biology [1]. Arguably, this is where the varying lack of consensus on a clear definition of IDDD may stem from [2]. The main clinical symptom of both DLSD and IDDD is low back pain (LBP), but not all IDD patients present with clinical symptoms [3]. LBP is a growing health problem in modern developed societies. Today, the term “diseases of affluence” is increasingly being used to refer to these disorders. LBP is an extremely common problem, but its extent is often trivialized [4]. About 26–42% of LBP cases are due to IDDD [5]. The natural course of IDDD is characterized by periods of exacerbation and remission of varying lengths. The patient’s fitness results from environmental factors, conservative treatment used, rehabilitation undertaken, and surgical procedures.

Population-based studies have shown that the annual incidence of a first LBP episode ranges from 6.3% to 15.4%, whereas the cumulative incidence of a first or any episode ranges from 1.5% to 36% [6]. The prevalence in the general population ranges from 1% to 58.1%, and the annual prevalence ranges from 0.8% to 82.5%. In the population of Great Britain between the ages of 20 and 59, the annual incidence is 36.1% [7]. One-year periods of remission occur in 54% to 90% of patients [4]. The average duration of pain in the course of LBP varies widely and can be 15.5 days for patients who experienced pain for <3 months and 128.5 days for those whose pain lasted for 3–6 months. Recurrences of subsequent episodes of LBP are noted in 50% of patients within the first year, in 60% within 2 years, and in as many as 70% within the first 5 years [8]. The recurrence rate has been shown to increase with age and to affect women more often [9].

Undoubtedly, LBP is the leading cause of reduced life activity and absenteeism from work worldwide and represents a huge economic burden on individuals, families, society and industry. Until recently, it was considered a problem mainly for highly developed countries; however, numerous studies have shown that it is just as serious in developing countries [10]. The economic costs associated with LBP are enormous and include medical care, compensation, benefits, lost productivity, worker retraining, and administrative costs. In Great Britain, it was estimated that direct and indirect spending related to LBP reached GBP 11 billion in 2000, one of the highest levels in the healthcare sector [11]. Similarly, in Australia, LBP was proven to be one of the costliest conditions, with direct and indirect expenses totaling AUD 9.14 billion in 2001 [12]. The hospitalization rates caused by LBP vary between 13.4% and 18.7%. Due to differences in methodological approaches, the annual total costs of USD 2.2 billion per population and USD 1226.25 per patient were documented for LBP [13].

In response to social and “market” demand, a variety of therapeutic methods have been developed, including pharmacological, rehabilitative and broadly defined treatment methods [14,15,16,17]. Surgical methods include transcanal access and intradiscal procedures. The former include laminectomy with discectomy and microdiscectomy. The main advantages of microdiscectomy are a good view of the surgical field and illumination with the use of a surgical microscope and a smaller skin incision. In turn, treatment outcomes do not differ between the two methods [18]. Intradiscal procedures include chemonucleolysis, automatic percutaneous lumbar discectomy with nucleotome, percutaneous endoscopic discectomy, intradiscal endothermal therapy and laser disc decompression [19,20]. In the ever-growing array of surgical treatment options, one of the most commonly performed procedures today is interspinous stabilization with a dynamic-interspinous-stabilizer-type DIAM (device for intervertebral assisted motion) [21].

Dynamic stabilizers, such as DIAMs, are able to absorb and dissipate axial loads and thus reduce the stresses acting on the intervertebral joints [22]. Another important effect resulting from the altered geometry of adjacent vertebrae, which has been demonstrated in numerous experimental studies, is the significant reduction in intradiscal pressure, especially in the posterior part of the intervertebral disc. The beneficial effect of intervertebral stabilizer implantation in patients with spinal canal stenosis and/or intervertebral foramen stenosis is largely due to the change in the geometry of the spinal canal and intervertebral foramina [23].

It seems very important to verify the effectiveness of surgical management with DIAM interspinous stabilization compared with less aggressive conservative and rehabilitative management. Currently, there is still no clear, strong evidence in the literature on the effectiveness and superiority of surgical treatment over conservative treatment, especially from a long-term perspective [24]. A systematic review showed that there are no differences between surgical and conservative treatment outcomes after one year and after 2 years; however, the quality of this evidence is very low [25]. Therefore, the primary goal of this study is to compare the early and long-term outcomes of DLSD patients treated with neurosurgical methods using the DIAM interspinous stabilizer with patients treated conservatively with rehabilitation procedures.

## 2. Materials and Methods

### 2.1. Study Design and Participants

The study was conducted in the Department of Neurosurgery at the Walbrzych Specialized Hospital (Poland). Nearly 80 surgical procedures for the neurosurgical implantation of the DIAM stabilization system in patients with DLSD were performed. Patients who did not return completed questionnaires one year after surgery despite being eligible for the study and treatment intervention were excluded from the analysis. This made it impossible to obtain complete data and follow-up observations for the treatment results. The final and complete study material consisted of 86 patients who were randomized to two comparison groups: A (*n* = 43), treated with the DIAM dynamic stabilization system for DLSD (mean age = 43.4 years ± SD = 10.8 years), and B (*n* = 43), treated conservatively for DLSD at a neurosurgical outpatient clinic at the same time (mean age = 41.4 years ± SD = 12.8 years).

### 2.2. Qualification Procedure

The inclusion criteria for patient participation in the study were: (1) moderate to severe low back pain as measured using the visual analogue scale (VAS) secondary to DLSD lesions at one L3-S1 level, (2) LBP defined as persistent back pain with or without root pain (sciatica), with a duration of the current pain episode of at least several months, (3) chronic LBP with sciatica, confirmed by magnetic resonance imaging (MRI) [26], (4) IDDD confirmed in patient history, physical examination and MRI, (5) no improvement after conservative treatment for at least 6 weeks and no more than 6 months before surgery, (6) informed consent given to participate in the study and (7) age over 18.

Contraindications to surgical treatment and at the same time criteria for exclusion from the study were: (1) reduction in disc height > 60% at the involved level, (2) inflammation of the arachnoid membrane, (3) primary diagnosis of the cause of spinal discomfort other than DLSD in the involved segment, (4) DLSD requiring the surgical treatment of more than one level, (5) history of conservative treatment within 6 weeks before the planned surgery, (6) sequestration of herniated nucleus pulposus, (7) history of any surgery of the involved or adjacent level, (8) history of any intradiscal ablation procedure, (9) congenital or iatrogenic absence of posterior column elements (e.g., condition after resection of the intervertebral joint, spondylolysis, fracture, closed spina bifida), (10) underdevelopment or agenesis of the spinous process, (11) lower limb movement deficit, (12) cauda equina syndrome, (13) clinically significant peripheral neuropathy, (14) chronic ischemia of the lower extremities manifested by intermittent claudication, (15) history of traumatic fracture in L1–L5 segments, either compressive or with tearing, (16) lumbar scoliosis with a Cobb angle greater than 15°, (17) established osteoporosis, osteopenia or osteomalacia, (18) obesity with a BMI of ≥40, (19) documented allergy to silicone, polyethylene, titanium or latex, (20) active bacterial infection, (21) active neoplastic disease or a history of neoplastic disease within the past 5 years including spinal neoplastic disease, (22) anesthetic contraindications to general anesthesia, and the lack of patients’ informed consent.

### 2.3. Surgical Treatment

Patients in the study group underwent surgical treatment with implantation of the DIAM stabilization system. During surgery, all patients were placed in the prone position. The DIAM stabilizer (Medtronic Spine and Biologics, Parkway, Minneapolis, MN, USA), which is a dynamic implant, is constructed of silicone covered with a polyester mesh and is held in place using polyester bands. Implantation of the stabilizer was performed under general anesthesia, with the patient in the supine position with the least possible pressure on the abdominal cavity. The patient was placed in a position that provided a slight flexion of the operated spine in order to increase the distance between the vertebral arches and increase the surgical approach field. The operating table allowed X-ray viewing using the C-arm. The paravertebral muscles were detached bilaterally from the spinous processes at the level of the intervertebral joints using a standard technique, with the continuity of the joint capsules of the intervertebral joints preserved. With the help of a dissector, the interspinous space from the supraspinous ligament to the yellow ligament was prepared. Only the elements of the intercondylar ligament and any osteophytes were removed, creating enough space for implant placement. This was performed with (Kerrison) bone forceps and punches. Proper debridement of the muscle and ligament attachments from the spinous processes allowed for proper positioning of the implant wings. At this stage, ligament fenestration was performed to decompress the dural sac. 

The distraction was gradually increased until the supraspinatus ligament and the joint capsules of the intervertebral joints were smoothed and the proper lordosis of the operated segment was achieved. The proper alignment of the joints and the spaces between the vertebral arches were controlled by fluoroscopy. If an abnormally large force was required to achieve distraction, or if resistance was felt at some point during distraction, the cause may have been the presence of pathological bone bridges between vertebral arches that should be removed. Once the mobility of the adjacent vertebrae was restored, distraction was proceeded by placing the distractor as close to the yellow ligament as possible. The goal of the surgery was to optimally decompress the operated spinal level, hence in some cases it was necessary to perform removal of part of the articular processes and widening of the intervertebral foramen. The DIAM stabilization system was then placed in place of the distractor to maintain adequate decompression, allowing the operated segment to remain mobile. 

Several gauges were taken with the measuring tools included in the DIAM kit, measuring 8 mm, 10 mm, 12 mm and 14 mm, to select the appropriate implant size. The largest fitting implant was chosen, avoiding excessive distraction that can cause kyphosis and, in extreme cases, lead to a fracture of the spinous process. The implant was placed in the applicator with its proper cephalocaudal orientation before it was implanted in the appropriate location. Two methods of implant placement were used. The first version used the temporary excessive space distraction, which greatly facilitates the introduction of the DIAM system. The DIAM system in the space was pushed with a special tool so that it was directly over the yellow ligament. Once the system was optimally placed, the distractor was removed. The second method of implanting the DIAM stabilization system used a temporary distraction–application clip. Once the system was inserted into the interspinous space, the clip was removed. The DIAM system was attached to adjacent spinous processes with tapes. As recommended by the manufacturer, no drain was used. Perioperative antibiotic therapy was administered according to standard preoperative and postoperative protocols.

### 2.4. Conservative Treatment

Patients in the control group received individualized conservative treatment to reduce pain and improve spinal function and quality of life without surgical intervention. Pain medication and spinal periarticular injections of anti-inflammatory drugs were administered. Depending on the severity of the pain, oral analgesics (NSAIDs) were recommended. Comprehensive rehabilitation activities were carried out, which were planned based on the SMART principle with a view to defining specific, measurable, achievable, relevant and time-bound goals for the therapy. Priority measures included reducing pain, reducing muscle stiffness and improving range of motion, enhancing muscle strength and endurance and improving soft tissue flexibility. Exercises aimed at improving postural stability and posture correction in standing and sitting positions were used. From the scope of manual methods, relaxing massage of the lumbar spine area and joint mobilization techniques of the lumbar spine (tractions and glides) were used. Of the physical therapy treatments, electrotherapy treatments using transcutaneous electrical nerve stimulation (TENS) were used. Patients were also made aware of their body’s limitations and capabilities in relation to the secondary prevention of LBP. Conservative treatment was applied for a period of 6 weeks and was conducted according to standards of the same rehabilitation team under the supervision of the same attending physician. Treatments were conducted 5 days a week and lasted 2 h a day. An educational program on proper postural habits and techniques for avoiding spinal overload and healthy lifestyles was also provided [27,28].

### 2.5. Outcome Measures

The following data, acquired prospectively, underwent evaluation: (1) selected demographic data, (2) presence of pain and other discomforts before, immediately after and one year after surgery according to the VAS scale, (3) degree of disability using the ODI questionnaire before, immediately after and one year after surgery, (4) duration of surgery measured from skin incision to suture, (5) occurrence of other discomforts and ailments after surgery. The patients independently completed the VAS scale assessment and ODI questionnaire: before the surgery and immediately after the surgery while in the hospital and one year after the surgery, receiving it by mail. The assessment was based on the returned completed questionnaires. The following contributions were made by the team: M.P.-B. played a key role in conservative treatment, A.D. was responsible for performing neurosurgery procedure, A.D., M.M. and B.C. handled data collection and M.M. and J.R. were responsible for reporting functional complaints.

#### 2.5.1. Pain Intensity

The VAS pain scale is one of the most popular tools for measuring the subjective intensity of pain or other somatic sensations. It consists of presenting a line, usually 10 cm long, on which the patient marks their subjective sensation. When measuring pain, the VAS scale usually has extreme descriptions: on one side 0, meaning no pain, and on the other side 10, meaning the highest possible pain. Measuring on the VAS scale is quick and easy and provides accurate and reliable results. In the case of pain, measurement on the VAS scale can be conducted by the patient alone or assisted by members of the medical staff, who ask the patient to mark the spot on the line that best corresponds to the intensity of their pain. The VAS scale is widely used in clinical, epidemiological and therapeutic studies to assess the effectiveness of treatment for pain or other somatic sensations, including those of spinal conditions. In the present study, we assessed pain intensity “right now” to capture the immediate, current perception of pain by the patients. For the statistical analysis and standardization of measurements, pain severity on the VAS scale was divided into 3 categories: I—0–4, II—5–6 and III—7–10. During the LBP evaluation, a location division was made in LBP with or without sciatica [29].

#### 2.5.2. Disability Level

The ODI questionnaire is a diagnostic tool that is used to assess the degree of disability associated with back pain. The ODI consists of 10 questions about daily activities such as getting out of bed, walking, sitting up, lifting objects, sleeping, doing housework and self-care. Each question has 6 possible answers that are scored from 0 to 5, where 0 means no difficulty in performing the activity and 5 means not being able to perform it. The points obtained in each question are added up and the final score ranges from 0 to 100, where 0 means no disability and 100 means total inability to perform daily activities. The higher the ODI score, the greater the patient’s disability [30]. 

#### 2.5.3. Functional Complaints

We also analyzed the occurrence and severity of other complaints reported by the patient, such as numbness in the limbs, stumbling, problems straightening the torso, problems emptying the bladder on neurogenic grounds, abnormal skin sensation of the perineal area and impotence.

### 2.6. Assessment Criteria

Criteria for evaluating the early success of surgical treatment: patients treated with surgery were observed in the early post-operative stage for improvement in terms of pain/disability as measured by the ODI, a difference in preoperative and post-operative scores of ≥15 points, no complications related to the DIAM stabilization system used or to the procedure and no need for reoperation during hospitalization.

Criteria of effectiveness of conservative treatment: patients treated conservatively were observed from the time for the decision not to undergo surgery, after 2 months of conservative treatment and one year after the beginning of follow-up. The criteria for treatment efficacy were identical to those for surgical patients.

Criteria for treatment efficiency: (1) reduction in disability as measured by the ODI, described as a difference in scores between the start of follow-up and follow-up control points of ≥15 points (a difference of <15 points was considered ineffective) and (2) elimination and/or reduction of back and/or lower extremity pain measured by the VAS described as difference in pain intensity > 0 (most severe pain before surgery—most severe pain after surgery, separately calculated for the back and lower extremities), lack of efficacy was considered if there was an absence of pain elimination or if pain became more intense (difference in VAS scores ≤ 0). The selection of a 1-point reduction in VAS scores as the criterion for clinical significance in our study, as opposed to the commonly employed 3-point benchmark, was guided by the specific context and objectives of our research. This decision was made to enhance the precision and applicability of our assessments, particularly in the early postoperative phase. The adoption of a 1-point reduction provides advantages in capturing nuanced changes in pain levels during this critical period following surgery.

### 2.7. Ethical Considerations

Approval from the Independent Bioethics Committee (IBC) at the Medical University of Wrocław was obtained (approval no. KB-136/2014). The study was conducted in accordance with the principles of good clinical practice (GCP), ensuring high quality, reliability and data security. During the study, the provisions of the Declaration of Helsinki (DoH) with regard to respecting patients’ rights were respected, in accordance with ethical and regulatory requirements. The Consolidated Standards of Reporting Trials (CONSORT) 2010 updated guidelines for reporting parallel group randomized trials were followed [31]. 

### 2.8. Sample Size

Before the commencement of the study, a sample size calculation was conducted using Statistica 10.0 (TIBCO, Software Inc., Palo Alto, CA, USA) to ensure the statistical validity and reliability of the research findings. Based on the analysis of prior research findings and clinical expertise, it was determined that the new intervention was anticipated to yield a medium effect size of 0.5 concerning pain reduction in comparison with the standard treatment. The significance level for this study was established at 0.05, denoting a 5% probability of committing a type I error (false positive). To ensure statistical robustness, a power of 0.80 was targeted, indicating an 80% likelihood of identifying a genuine effect should it be present, thus mitigating the risk of a type II error. A two-tailed test was selected to enable the detection of any significant difference, irrespective of whether it manifested as an increase or decrease in pain. Following these considerations, it was determined that a total sample size of 80 individuals was required, with 40 participants allocated to each of the two groups under investigation.

### 2.9. Statistical Analysis

The study included statistical analyses on the relationship between various variables and the effectiveness of the treatment and the reduction in back and lower extremity pain. Age, duration of hospitalization and surgery time were described using the arithmetic mean, standard deviation and minimum and maximum measurements. The severity of pain on the VAS scale was divided into three categories: category I—0–4, category II—5–6 and category III—7–10. Statistical tests, including the chi-squared test with Yates’ correction, Fisher’s exact test, the Fisher–Freeman–Halton test, and Pearson’s chi-squared test, were used in the analyses. The statistical software used in the study was Statistica 10.0 (TIBCO, Software Inc., Palo Alto, CA, USA) and CytelStudio 10.0 (CytelStudio Software Corporation, Cambridge, MA, USA). The study was conducted at a significance level of α = 0.05.

## 3. Results

### 3.1. Participants’ Characteristics

The study included 86 patients, of whom 43 patients (mean age 43.4 ± 10.8 years) underwent neurosurgical treatment with the DIAM interspinous stabilization system and 43 patients (mean age 41.4 ± 12.8 years) were treated conservatively at a neurosurgical outpatient clinic at the same time (Table 1). 

### 3.2. Back Pain

**Figure 1:**
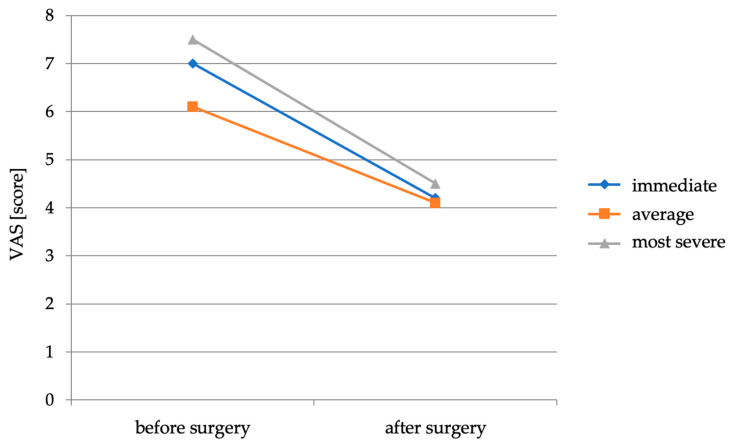
Average back pain intensity scores on the VAS scale before and immediately after surgery.

All of the 43 patients who experienced LBP before surgery continued to experience it after surgery. On average, the intensity of the pain experienced immediately after the procedure was 4.2. The average intensity of the pain experienced during the postoperative period was 4.1. The most severe back pain experienced in the postoperative period averaged 4.5. The average VAS back pain intensity scores before and immediately after the procedure are shown in Figure 1. 

Pain relief in the form of reduced overall LBP intensity occurred in 32 patients (74.4%). Thirty-one patients experienced a reduction in perioperative discomfort (shortly before and after surgery), six had no improvement in this area and six experienced an increase in pain. Twenty-five patients experienced a decrease in the average intensity of the pain felt, five patients had no change in the intensity of pain and nine patients experienced an increase in the average intensity of pain. In 33 patients there was a reduction in the discomfort described as the most severe one experienced by the patient, in 5 patients its intensity did not change and in the remaining 5 there was an increase in the severity of pain. On average, there was a decrease in the severity of pain experienced immediately before and after the procedure by 1.4 points on the VAS scale; the average pain experienced decreased by 2.1 points and the most severe pain experienced decreased by 1.5 points.

### 3.3. Lower Limbs’ Pain

**Figure 2:**
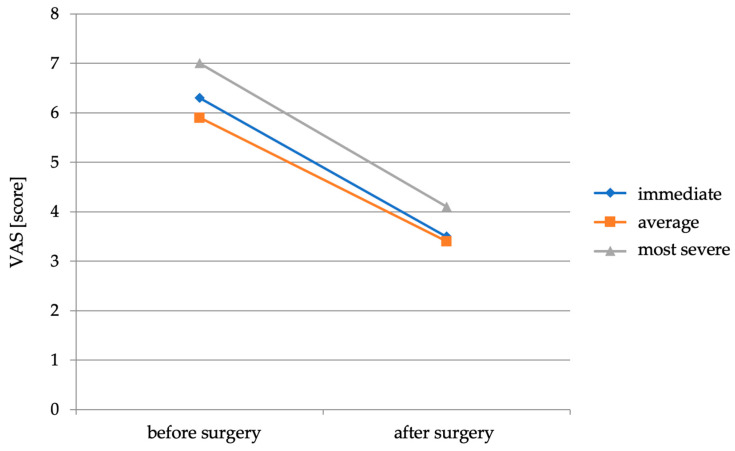
Average lower limb pain intensity scores on the VAS scale before and immediately after surgery.

Of the 36 patients who experienced lower extremity pain before surgery, all continued to experience lower extremity pain associated with sciatica in the postoperative period. On average, the intensity of the lower extremity pain experienced immediately after the procedure was 3.5 points. The average intensity of pain experienced in the postoperative period was 3.4 points. The most severe back pain experienced in the postoperative period averaged 4.1 points. The mean VAS lower extremity pain intensity scores before and immediately after the procedure are shown in Figure 2.

It was found that 29 patients (80.5%) experienced a reduction in LBP with sciatica pain (difference in VAS scores before and after the procedure). In 29 patients there was a reduction in the discomfort experienced in the period immediately after the procedure, 5 patients showed no improvement in this regard and 3 patients experienced an increase in pain. Twenty-nine patients experienced a decrease in the patient’s average pain intensity, three experienced no change in their average pain intensity and four experienced an increase in their average pain intensity. Twenty-nine patients experienced a decrease in LBP with sciatica described as the most severe pain experienced by the patient, five patients had no change in pain intensity and the remaining two patients experienced an increase in pain intensity. Four patients did not improve in one or two types of pain, whereas the other five did not improve in terms of the severity of all three types of pain. In one patient (outside the analyzed group of 36 patients with LBP with sciatica before surgery), a root syndrome from sciatic nerve compression appeared only after surgery. On average, there was a decrease in the severity of LBP with sciatica felt immediately after the procedure by 2.6 points on the VAS scale, the average pain felt decreased by 2.3 points and the most severe pain felt decrease by 2.6 points compared with the same parameters studied before the procedure. 

### 3.4. Disability Level

**Figure 3:**
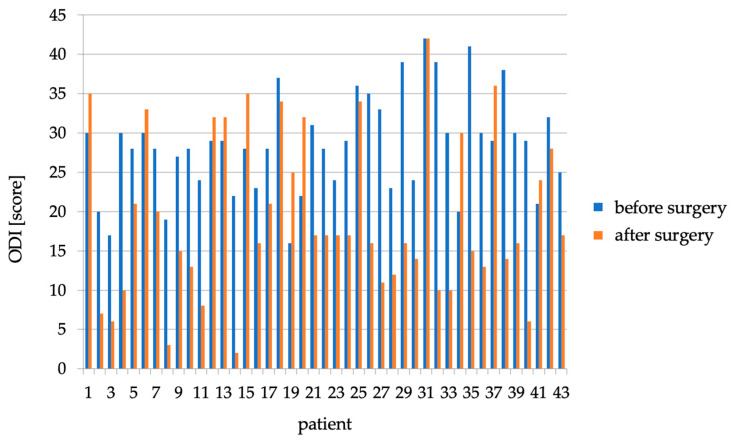
ODI scores before and immediately after one year of surgery for individual patients.

The average score was 19.3 ± 10.3 points. The difference in ODI questionnaire scores (total scores before treatment—total scores after treatment) averaged 9.1 ± 10.6 points. Fourteen patients had a decrease in score of ≥15 points (32.6%), eighteen patients had a decrease in score from 1 to 14 points (41.9%), one patient had the same number of points before and immediately after the procedure (2.3%) and ten patients (23.2%) had an average increase of 6 points (from 3 to 10 points) (Figure 3).

**Figure 4:**
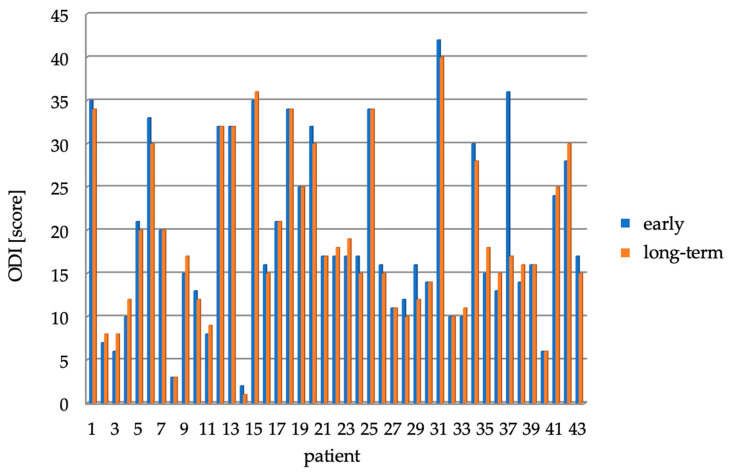
ODI scores right after surgery and after one year for individual patients.

Compared with scores on the ODI questionnaire immediately after surgery, one year after surgery there was an average decrease in score of 0.4 ± 3.3 points (the maximum decrease was 19 points, whereas the maximum increase was 3 points, median 0). There was a decrease of 19 points in one patient, a decrease of 4 points in one patient, a decrease of 3 points also in one patient, a decrease of 2 points in five patients, a decrease of 1 point in six patients, scores did not change in 14 patients, six patients had an increase of 1 point, eight patients had an increase of two points and one patient had an increase of 3 points. A graphical representation of the difference in scores just after surgery and one year after surgery is shown in Figure 4. Remarkably, none of the 43 patients required reoperation within 12 months of surgery.

### 3.5. Surgical vs. Conservative Treatment

Based on the adopted criterion of improvement according to the ODI (an improvement was considered to be at least a 15-point reduction in complaints), the results for the surgical patients and the patients treated conservatively for 2 months (early outcome) were compared. In the study group, improvement according to the accepted criteria was achieved in 14 patients (32.5%), whereas in the control group it was achieved in 15 patients (35%). The two groups were also compared one year after treatment (long-term outcome). In the study group, the number of patients with improvement did not change. In the control group, 16 patients (37%) showed improvement after one year. Statistical analysis using the chi-squared test showed no statistically significant differences between the two groups in both early (*p* = 0.45) and long-term (*p* = 0.37) disability reduction outcomes. The results are presented in Table 2.

## 4. Discussion

The study presented here was designed to demonstrate whether neurosurgical treatment with the DIAM interspinous stabilizer was more effective than conservative management, including rehabilitation. Within the framework of the present study, it was decided to verify the following hypotheses: (i) DIAM stabilizer treatment has a more effective analgesic effect immediately after surgery than conservative treatment after 2 months; (ii) DIAM stabilizer treatment has a more effective analgesic effect at 1 year after surgery than conservative treatment after 1 year; (iii) among the demographic and clinical variables analyzed, there are factors that give a better prognosis after surgery; and (iv) better results are obtained in the treatment of LBP without sciatica than LBP with sciatica in surgical patients. 

The results show that patients (*n* = 43) who experienced LBP without sciatica before surgery continued to experience back pain after surgery (mean VAS score of 4.2). Of the 36 patients who experienced lower extremity pain before surgery, all continued to experience pain in the postoperative period (mean score of 3.5). In 29 patients (80.5%), there was a reduction in LBP with sciatica. The mean score on the ODI questionnaire at follow-up was 19.3 ± 10.3 points. The difference in ODI questionnaire scores in the pre-treatment vs. post-treatment results was 9.1 ± 10.6 points on average. At 12 months after surgery, none of the patients (*n* = 43) required reoperation. There were no statistically significant differences regarding disability between the two groups in both early (*p* = 0.45) and long-term (*p* = 0.37) outcomes using the ODI questionnaire, demonstrating the equal effectiveness of surgical and conservative treatments.

In the current literature one can find quite a number of works relating to the research topic under discussion. Sobottke et al. [32] analyzed the radiological data of 129 patients who were implanted with one of three stabilizers (X Stop, Wallis or DIAM). The posterior and anterior height of intervertebral discs, intervertebral angle and width, height and area of intervertebral foramina were analyzed. Significant changes in the abovementioned parameters were shown after surgery compared with the preoperative state—there was an increase in the dimensions of all examined elements except the intervertebral angle, which decreased. However, during the follow-up period (which ranged from 240 to 1494 days), all of the aforementioned parameters returned to pre-surgery dimensions. The study showed a statistically significant improvement in pain complaints as assessed by VAS. Interestingly, although the radiological parameters returned to their preoperative state over time, the patients’ pain symptoms did not increase. The decrease in pain was shown to correlate slightly with the parameters on imaging studies (*p* < 0.05, r = 33). 

The goal of LBP treatment is to reduce pain and thus improve quality of life. In the present study, a difference in ODI questionnaire scores of 15 or more, representing a minimum clinically important difference (MCID), was used as a criterion for treatment efficacy. Its concept is proposed as a standard for evaluating the effectiveness of a given treatment method and in describing the patient’s satisfaction regarding a given method. In the literature, one can find different MCID values for the ODI—from 10.0 to 16.3 points [33]. The MCID in pain reduction on the VAS before and after surgery (both for LBP with and without sciatica) in this study was taken as 1 point. The literature also includes 2 points as the MCID value for VAS [34]. 

In the present analysis, treatment was successful in only 32.6% (14 patients); the remaining patients did not meet the criteria for efficacy, which was considered at least a 15-point improvement in ODI score. Among these remaining 29 patients, 18 (41.9%) had a decrease in ODI scores not exceeding 14, one patient had no change in scores and 10 (23.2%) patients had an increase in scores (from 3 to 10 points, with an average of 6). Compared with the scores on the ODI questionnaire collected just after surgery, one year after surgery there was an average decrease in score of 0.4 ± 3.3 points (the maximum decrease was 19 points, whereas the maximum increase was 3 points). Therefore, the treatment outcome remained the same during the one-year follow-up period. Heterogeneous treatment efficacy criteria do not allow direct comparison of the results obtained in this analysis with those of other researchers. 

In the studies conducted to date on DIAM stabilization, improvements have been achieved in selected groups of patients. In a previously cited paper, Ha et al. [35] analyzed the radiological and clinical outcomes following DIAM implantation in 31 patients. There were no significant differences in the radiological images of the studied features (among others disc height, cross-sectional area and height of intervertebral foramina) outside the range of motion in the segment behind the instrument. However, there was a clinically significant improvement in the ODI and VAS scores (*p* < 0.001). There was no correlation between intervertebral disc height on postoperative imaging studies and ODI score (*p* = 0.46). This study failed to link the clinical improvements with the changes in the radiology images and therefore confirm the mechanism of indirect intervertebral disc decompression.

Buric et al. [36] are among the few authors to present very good results for 53 patients who underwent surgery using the DIAM system. The treatment was considered effective, taking a Roland–Morris disability questionnaire (RMDQ) change ≥30% as a criterion for success; this was achieved in as many as 46 (88%) patients. In another study, the same research team analyzed the long-term outcomes of 52 patients. A statistically significant reduction in RMDQ disability score was observed at 2 months (8.6, 95% CI 7.4; 9.9) and at 48 months after surgery (7.5, 95% CI 6.1; 8.9) compared with the condition from before surgery. The mean scores at follow-up examinations 2, 6, 12, 24 and 48 months after surgery were similar (*p* = 0.005). Analysis of the results in the groups separated per-protocol (PP) also showed a statistically significant (*p* < 0.0001) reduction in disability at 2 months (9.1, 95% CI 7.8; 10.4) and at 48 months after surgery (8.0, 95% CI 6.5; 9.4) compared with before surgery. The mean scores were similar (*p* < 0.1934) in the months between 2 and 24 but significantly increased in the 24-month to 48-month follow-up period (−2.1, 95% CI −3.5; −0.7, *p* = 0.0216). The percentage of patients who achieved MCID (≥30% of RMDQ scores) between pre-treatment and 48-month follow-up was 78.9% in the ITT population and 80.8% in the PP population. Unlike in our study using the ODI questionnaire, their study used the one by Buric et al. [36], i.e., the RMDQ works better for patients with minor disabilities. The good results obtained by the Italian researchers may mean that the patients who qualified for the study had a better prognosis anyway due to their lesser disability at baseline. For this reason, it is difficult to make a clear comparison between the results of the two research projects.

Mariottini et al. [37], in their 2005 paper analyzing the initial results of treatment with the DIAM implant in 43 patients, used Henderson’s failure classification to assess efficacy. The results were divided into four classes, where class 1—excellent result—means no pain and no restrictions on the activities of daily living; class 2—good result—means occasional pain (<12 h), the possibility of returning to pre-surgery activities and minimal restrictions on physical activity; class 3—medium result—means pain requiring relief, a reduction in professional activity and restriction of physical activity; and class 4—bad result—means complaints as before surgery, no possibility of professional activity and need for continuous analgesic drug treatment. Based on the assessment of functional improvement conducted with a questionnaire among patients after the procedure in the cited study, the outcome of 18 patients (44%) was rated as class 1, 22 patients were rated as class 2 (53%) and 1 patient was rated as class 3 (2.3%). To sum up, as many as 97% of patients rated the outcome of treatment as satisfactory. However, the authors do not report the duration of the time period over which the results were collected, i.e., whether it was a short period after surgery or in follow-up studies that took place from 12 months to 5 years, with an average of 34 months, or whether these results were maintained during the follow-up period. Moreover, the analyzed patients did not have DIAM implantation alone but also underwent discectomy or multilevel hemilaminectomy; it was also not stated how many patients underwent these procedures. A single implant was placed in 31 patients, whereas the remaining patients had two implants or more. The results of the cited work are quite unreliable. Nevertheless, the authors make a very bold conclusion based upon them—they assume that it is always better to place a DIAM when there is doubt about the surgical treatment of back pain. What is more, they say that there are no consequences from implanting the DIAM system even when the patient is inadequately qualified for the procedure and that it would be worse not to place the implant in a patient with instability. Unlike the analysis by Mariottnini et al. [37], the present study focused on patients who underwent surgery with the DIAM stabilizer only and did not include patients who also simultaneously underwent other surgeries. 

Similarly, a Czech study of 68 patients with LBP used the DIAM system alongside discectomy or foraminotomy and facetectomy [38]. The VAS was used to assess pain intensity, the ODI was used to determine disability and Odom’s classification was used to assess patient satisfaction with treatment. The control period was between one and three years. The authors’ average ODI score multiplied by 2 and expressed as a percentage was 60.44% before and 21.85% after the procedure, giving an average improvement of 63.85%. In the respective subgroups, the improvement in ODI scores was as follows: in patients with intervertebral disc herniation—an average of 39.62%, in patients with recurrent intervertebral disc herniation—41.5%, and in patients treated for stenosis of the intervertebral foramina—39.79%. In addition, patients who had the implant placed at the L3/L4 level recorded the greatest improvement (48% ODI). Patients who underwent discectomy had an improvement of 40% in ODI score, whereas those who underwent foraminotomy and partial facetectomy demonstrated an average improvement of 32.89%. The outcome of treatment according to Odom’s criteria, assessed 3 years after the procedure, was as follows: excellent—41% of patients, good—51.5%, and satisfactory—7.5%. The results of the cited paper are also very enthusiastic. However, it should be noted that every patient in this study underwent discectomy (patients with intervertebral disc herniation) or foraminotomy (patients with stenosis of the intervertebral foramina). Thus, similarly to Mariottnini et al. [37], the result obtained may be due only to the decompression of nerve structures and not due to the implant. In the present analysis, efficacy was achieved in only 32.6% of patients. However, it should be noted that it was only the implant that was placed in the patients, with no other procedures such as discectomy. Hence, the conclusion is that the stand-alone application of the DIAM system has little effectiveness. To determine the effectiveness of the DIAM system as an additional tool for improving the surgical outcomes of decompressed structures such as foraminotomy, partial facetectomy, fenestration or flavectomy, studies comparing the effectiveness of these procedures with or without the implant would need to be conducted. 

Kim et al. [39] conducted a study on a group of 62 patients. Half of these patients underwent microdiscectomy and/or hemilaminectomy with the DIAM system, whereas the other half underwent the aforementioned procedures without stabilizer implantation. Indications for the implantation of the DIAM stabilizer in the cited study were recurrent intervertebral disc herniation with worsening LBP, massive intervertebral disc herniation with root pain and significant LBP and spinal canal stenosis with instability. The VAS was used to assess pain intensity and the modified Macnab scale, described above, was used to assess treatment outcome. All patients in both groups showed a statistically significant improvement in pain complaints as assessed by the VAS. However, there was no significant difference between the two groups of patients. There were also no significant differences between the group of patients treated with the DIAM system and the group without the stabilizer in terms of satisfaction with the procedure, as assessed using Macnab criteria. Moreover, there were no significant differences in the radiologically evaluated anterior and posterior intervertebral disc heights, the distance between vertebral arches or the spondylolisthesis correction. Hence, the conclusion that the use of the DIAM system as an addition to decompression surgery does not improve patient outcomes. These results appear to be consistent with those presented in the present study, where the improvement achieved with the DIAM stabilizer was comparable with the use of conservative treatment alone. It should be noted that any surgery, including the use of a DIAM stabilizer, implies the need for post-operative drug treatment and rehabilitation, thus bringing the two groups of patients together even further. The only noticeable difference in the management of the study group is the implantation of an interspinous stabilizer, which did not improve patient outcomes in the present study.

Similar results were obtained in the work of Richter et al. [40] on a group of 60 patients with spinal canal stenosis, 30 of whom underwent a decompression procedure (partial laminectomy, removal of the yellow ligament or facetectomy), whereas the other 30 underwent a decompression procedure using the Coflex^TM^ system. The evaluated factors included pain severity on the VAS, the ODI scale, the degree of disability on the RMDQ questionnaire, the pain-free walking distance and a subjective assessment of satisfaction with the procedure. Statistically significant (*p* < 0.001) improvements were obtained in all clinical parameters (VAS, RMDQ, ODI, walking distance and subjective satisfaction) in both groups. There were no significant differences between the two groups. At the two-year follow-up, the results were similar—there were no significant differences between the two groups in terms of the outcomes assessed. Here too, the conclusion is that a stabilizer as an addition to decompression surgery in spinal canal stenosis does not improve treatment outcomes. 

A systematic review and meta-analysis by Wu et al. [41] demonstrated that there were no significant differences in the clinical outcomes of patients treated for spinal canal stenosis between the group treated with standard decompression methods and the group treated with interspinous stabilizers. The cited meta-analysis did not list the respective stabilizers. The meta-analysis by Moojen et al. [42] evaluated whether the procedure with implantation of an interspinous stabilizer is more effective than classic decompression surgery in patients with neurological claudication in spinal canal stenosis or at least more effective than conservative treatment (e.g., corticosteroid injections). The analysis included 11 studies, including three randomized trials and eight cohort studies. It did not specify whether the implantation of stabilizers was an additional or the sole procedure. In their conclusions, the authors claim that surgical decompression accompanied by implantation of an interspinous stabilizer is superior to conservative treatment. However, they stress that the quality of the evidence is poor. However, there is no answer to the question of whether stabilizer implantation alone is superior to a classic decompression procedure.

A large meta-analysis presented by Kaye et al. [43] covered 1575 patients; the DIAM system was implanted in 1315 of them, whereas the remaining 260 patients received the Aperius PercLID system (Medtronic, Operational Headquarters, Medtronic Parkway, Minneapolis, MN, USA). The indications for insertion of the DIAM system were degenerative disc disease (478 patients), stenosis of the intervertebral canal and/or orifices (in 347 patients), intervertebral disc herniation (in 283 patients) and intervertebral joint pain syndrome (143 patients). The Aperius system was used in elderly patients with multiple comorbidities, as its implantation procedure could be performed under epidural or local anesthesia. The Zurich claudication questionnaire (ZCQ), the EuroQol 5 domain questionnaire (EQ-5D) and the VAS were used to assess the pre- and post-operative conditions. The end result of treatment was evaluated according to the Macnab criteria. Follow-up examinations were performed 2, 6 and 12 months after the procedure. Statistically significant improvements were noted in all the parameters used. An excellent result was obtained in 58.7% of patients, a good result in 30.9% of patients, a satisfactory result in 6.2% of patients and a poor result in the remaining 4.2% of patients. The results were not distinguished according to the system used. However, in that study the DIAM system was also used in addition to the primary decompression procedure or discectomy. 

In turn, a 2022 meta-analysis by Chiou et al. [44] covered 17 RCTs and retrospective studies that included 1255 participants. Topping-off intervertebral implants (DIAM; OR = 0.235, *p* < 0.001), Dynesys (OR = 0.413, *p* < 0.001) and Coflex (OR = 0.417, *p* < 0.01) significantly reduced the incidence of radiographic adjacent segment degeneration (RASDeg) compared with spinal stabilization surgery alone. Spinal stabilization supplemented with DIAM significantly reduced the incidence of clinical adjacent segment disease (CASD) (OR = 0.358, *p* = 0.032). Spinal stabilization supplemented with DIAM significantly reduced the incidence of radiographic and clinical adjacent segment disease. No significant differences were observed between the comparison groups with regard to reoperation for adjacent segment disease (ASD) and back pain relief scores.

The previously cited analysis by Sobottke et al. [32] covered 129 patients who were implanted with one of three stabilizers (X Stop, Wallis or DIAM) due to spinal canal stenosis. The procedure consisted of implanting one of the three stabilizers without surgical decompression. The DIAM implant was placed in 25.6% of patients (33), the X Stop in 60.5% of patient and the Wallis implant was placed in 14.0% of patients. VAS scores (determined in the 0–100 range) decreased statistically significantly compared with the pre-treatment condition. The improvement was significantly maintained throughout the entire follow-up period (averaging 527 ± 377 days). In the group of patients who were implanted with DIAM, the average VAS score before the procedure was 59.5 ± SD 23.2; after the procedure it fell by an average of 34 ± 34.3, and at the first follow-up (averaging 202.3 ± 231.9 days) it fell by an average of another 2.6 ± SD 41.9. There were no significant differences in VAS scores between the groups of patients with each stabilizer, although the group of patients with the DIAM system in place showed a trend (*p* = 0.083) toward better results than the group of patients with the Wallis system in place. In addition, there was no correlation between age and change in VAS score after surgery. Significantly higher VAS values were found in the preoperative evaluation in the women (*p* = 0.018); however, analysis of the VAS differences after surgery showed no significant gender dependence.

In order to better distinguish the benefits obtained from the procedure, the present study separately analyzed LBP without and with sciatica in the course of the sciatic nerve compression. This separation makes it possible to determine the degree of impact of the procedure used on both discogenic pain and root pain. Pain relief in the form of a reduction in the intensity of overall back pain occurred in 32 patients (74.4%). On average, there was a 1.4-point decrease in the severity of pain experienced immediately before and after the procedure on the VAS scale. Pain relief in the form of reduced overall LBP with sciatica was achieved in 29 patients (80.5%). On average, there was a 2.6-point decrease in the severity of pain experienced immediately before and after the procedure on the VAS.

In the study by Burica et al. [36] on a population of 53 patients with an implanted DIAM, the mean VAS score before surgery was 6.0 ± 1.9. The mean VAS score decreased by 1.9–2.9 at follow-up examinations conducted between 2 and 48 months after the surgery. A statistically significant increase in scores was observed between months 24 and 48, but the mean score was still significantly lower at month 48 compared with the pre-surgery status. In the cited work, 1.2 and 1.5 points were adopted for the MCID. In the entire study population, 71.2% of patients recorded an MCID of 1.2 points, whereas 67.3% of patients recorded an MCID of 1.5 points between their preoperative condition and 48 months after surgery. In the study by Fabrizi et al. [45] on a population of 1575 patients provided with the DIAM or the Aperius system, the mean VAS score dropped from 7.54 ± 2.14 points to 2.41 ± 1.78 points. In that trial, the mean VAS score of back pain before surgery was 6.11 points and the mean score of maximum back pain experienced was 7.5 points.

In a Czech study (*n* = 68), the average improvement in VAS score was 70.75% (from 7.19 points before surgery to 2.10 points after surgery). In individual subgroups, the differences in VAS before and after surgery were as follows: patients with intervertebral disc herniation experienced average drops of 5.42 points, patients with recurrent disc herniation—drops of 5.00 points and patients with intervertebral foramen stenosis—drops of 5.18 points. In patients after discectomy the decrease averaged 5.17 points, whereas after foraminotomy and partial facetectomy the decrease was 4.78 points. It should not be forgotten that in the cited work, the DIAM was always used in addition to the decompression procedure. In our study, the average decrease in VAS scores was 2.1 points for back pain and 2.6 points for lower extremities. However, in all patients, the reported decrease in pain intensity was the result of stabilizer implantation without a classic decompression procedure. 

The study by Taylor et al. [46] was conducted in three French centers and involved 104 patients. In 7 of these patients, the DIAM implantation was an isolated procedure, whereas in the remaining 97 patients it was performed as an additional procedure to discectomy, foraminotomy, laminectomy and hemilaminectomy and facetectomy. The purpose of the analysis was to verify the effectiveness of the DIAM implantation in modifying the intensity of pain experienced by the patient. Based on the questionnaires returned by the patients at the 6th month after the procedure, there was a decrease in pain medication intake in 50% of the patients, an increase in pain medication intake in 18.2% of the patients and no change in 31.8% of the cases. On average, 18 months after surgery, 63.1% of patients said they had reduced their pain medication intake. During this period, pain persisted in 85.3% of patients (back pain in 78%, lower extremity pain in 72%). Complete pain relief was observed in only 14.7% of the cohort. Declarations of the amount of pain medication intake are somewhat at odds with the improvement in pain after surgery presented by the authors—a decrease in pain should be associated with a decrease in pain medication intake. Thus, a decrease in pain levels, recorded by the physician, occurred 88.5% of patients, whereas only half of the patients reduced their doses of analgesics and more than 30% declared taking the same amount of medication. 

Noteworthily, there was one intraoperative complication in the present trial, namely damage to the dural sac and leakage of cerebrospinal fluid. In addition, three patients (6.9%) developed proprioception disturbances after the procedure, in the form of difficulty in assuming an upright posture, and one patient developed stumbling. Two patients developed a problem with erecting their torso (5.4%), one patient reported a problem with micturition (2.4%), one patient reported a problem with abnormal skin sensation of the perineal area (2.4%) and one patient reported impotence after the procedure (7.7%). In addition, postoperative wound pain was reported by 30 patients (69.8%) after surgery, a long hospital stay was annoying for 12 patients (27.9%), a long incapacity for work was annoying for 4 patients (9.3%) and 14 patients (32.5%) found the postoperative scar annoying. 

Such a low number of complications is due to the fact that the DIAM system implantation procedure is simple for an experienced neurosurgeon. The procedure is short (in the present study it averaged 39.4 min, ranging from 20 to 90 min, median 35 min), does not involve much blood loss and does not require special perioperative care. The patient can be mobilized within a short time after the procedure—as early as on the first day. These are huge advantages for this method that speak in favor of its use. The patient usually tolerates the procedure well and does not require long hospitalization.

In the present study, no patient underwent reoperation during the one-year follow-up period. Follow-up is completed 12 months after the surgery and there are no data on reoperation (surgery of the segment on which the DIAM was placed) after this period. Given the fairly low success rate of the procedure, the likelihood of reoperation in subsequent years is high. It has been demonstrated that 8% of patients who underwent decompression surgery with implantation of the DIAM system due to spinal canal stenosis or intervertebral disc herniation will undergo reoperation within 4 years after the procedure. In the cited analysis, the reasons for reoperation were recurrent canal stenosis or intervertebral disc herniation, postoperative spondylolisthesis and long-term wound infections [47]. The risk factor for reoperation was the procedure at the L5/S1 level. In the available literature, the reoperation rates range from 2.5 to 11.5% [36,45]. In another study, the indispensability of reoperation was also the result of growing pain [36]. These patients underwent intervertebral joint thermoablation or arthrodesis. In one case, there was mechanical damage to the DIAM due to a fall, so the implant was replaced. In the study by Fabrizi et al. [36], the reoperation rate was 2.5% (in 40 of 1515 patients). The reason was the lack of efficiency in treating severe back pain. Ultimately, 8 of these patients underwent PLIF, 22 underwent spondylodesis with transpedicular stabilization and the remaining 10 underwent intervertebral disc removal. In another study analyzing 52 patients for whom the DIAM implantation was an isolated procedure, the need for reoperation arose in 4 patients (7.7%) [48].

### 4.1. Study Strengths

The present study stands out from the others in the available literature for its analysis of treatment outcomes using the DIAM system because of its relatively stringent efficacy criteria, homogeneous study group and adequately long follow-up period. Both groups of patients were recruited under the same conditions, at the same time and on an equal basis. The only difference was the patient’s own decision on further treatment. In addition, all patients were operated on by the same surgeon and treated conservatively by the same rehabilitation team. This approach has yielded results that do not share the enthusiasm of some of the cited authors but remain consistent with large analyses focused on the desirability of using the interspinous stabilizer alone. Compared with microdiscectomy, discectomy or decompressive laminectomy, the method of interspinous stabilization is relatively recent. Because classic surgeries have been performed for many years and are well known to every neurosurgeon, comparing new methods with them may be not meaningful. Although the implantation of the stabilizer itself is technically quite simple, the correct qualification of the patient for surgery requires experience, of which, in the case of this relatively new method, there is incomparably less than with the classical methods. Thus, it seems reasonable, as applied in the study, to compare the results of surgical treatment with conservative treatment, rather than with the other surgeries used. 

### 4.2. Study Limitations

This study has some potential methodological limitations to be discussed. Including only patients undergoing isolated DIAM implantation in the study inevitably entails a significant reduction in the number of subjects studied. This procedure is considered by most surgeons as an adjunct to surgical methods with long-established efficacy. There may have been confounding factors such as psychological issues in the selection of both groups, the impact of which on treatment outcomes cannot be accurately assessed. Finally, during the observation period, only the DIAM implant was used for open interspinous stabilization. The choice of only one type of implant, on the one hand, allowed an accurate assessment of its effectiveness but, on the other hand, it may have been a suboptimal decision in some cases. In an era of very rapid development of interspinous stabilization methods, a larger array of implants will perhaps allow for more precise customization and better treatment results.

Although our study used a clinical (physical examination) and radiological (MRI) classifications for discogenic low back pain (LBP), it is important to acknowledge the limitations of this approach. In future studies, the incorporation of widely recognized classifications, such as the Quebec task force (QTF) classification, may offer valuable insights. The QTF classification provides a standardized framework for categorizing LBP patients, which can enhance the comparability of research across different institutions and facilitate a more comprehensive understanding of the condition. Utilizing such established classifications in future investigations could contribute to a more in-depth analysis of patient profiles, outcomes and treatment strategies, ultimately advancing our understanding of discogenic LBP and optimizing patient care.

## 5. Conclusions

Neurosurgical treatment with the DIAM interspinous stabilizer was as effective as conservative treatment and rehabilitation during the follow-up period. Patients who had predominantly LBP rather than LBP with sciatica benefited more from the procedure. There is still a need for well-designed studies comparing medical procedures, both surgical and conservative, in the treatment of spinal pain in adults. 

## Figures and Tables

**Table 1:** Characteristics of study participants.

Characteristic	Study Group (*n* = 43)	Control Group (*n* = 43)
Women/men	22/21	22/21
Age (years)		
range	24–65	21–68
mean ± SD	43.4 ± 10.8	43.4 ± 10.8
BMI (kg/m^2^)		
range	18.5–30.2	22.1–31.9
mean ± SD	24.6 ± 4.1	26.7 ± 3.2
LBP duration (months)		
range	4.0–202.1	4.5–185.3
mean ± SD	55.7 ± 50.1	60.8 ± 52.9
Duration of sciatica (months)		
range	2.7–63.5	3.5–75.4
mean ± SD	14.2 ± 9.7	17.4 ± 10.3

**Table 2:** Early and long-term treatment outcomes in the study and control groups using the ODI questionnaire.

Observation Period	Percentage of Improvement in the Study Group	Percentage of Improvement in the Control Group	*p*-Value *
Early result	32.5%	35%	*p* = 0.45
Long-term result	32.5%	37%	*p* = 0.37

* Chi-squared test.

## Data Availability

The authors confirm that all data underlying the findings described in this manuscript are fully available to all interested researchers upon request.
